# Predominance of Antioxidants in Some Edible Plant Oils in Ameliorating Oxidative Stress and Testicular Toxicity Induced by Malathion

**DOI:** 10.3390/life12030350

**Published:** 2022-02-28

**Authors:** Isam M. Abu Zeid, Khalid M. Al-Asmari, Hisham N. Altayb, Atef M. Al-Attar, Safa H. Qahl, Mohammed Y. Alomar

**Affiliations:** 1 Department of Biological Sciences, Faculty of Science, King Abdulaziz University, P.O. Box 80203, Jeddah 21589, Saudi Arabia; ialmuan@kau.edu.sa (I.M.A.Z.); alattar@kau.edu.sa (A.M.A.-A.); myalomar@kau.edu.sa (M.Y.A.); 2 Princess Dr. Najla Bint Saud Al-Saud Center for Excellence Research in Biotechnology, King Abdulaziz University, P.O. Box 80200, Jeddah 21589, Saudi Arabia; 3 Department of Biochemistry, Faculty of Science, King Abdulaziz University, Building A 90, Jeddah 21589, Saudi Arabia; hdemmahom@kau.edu.sa; 4 Department of Biology, College of Sciences, University of Jeddah, Jeddah 21959, Saudi Arabia; shqahal@uj.edu.sa

**Keywords:** malathion, plant oil, testicular, antioxidant, in silico

## Abstract

Malathion (MAL) is an insecticide that has been linked to reproductive system damage in both humans and animals. In the present investigation, the antitoxic effects of coffee and olive oils on MAL-induced testicular dysfunctions were evaluated. MAL-intoxicated rats were supplemented with coffee and olive oils (400 mg/kg) for 7 weeks. Exposure to MAL resulted in statistically altered antioxidant enzymes and histopathological findings of necrotic seminiferous tubules and spermatogenetic arrest in rats after seven weeks of treatment. The effects of MAL intoxication on physiological and histopathological changes were improved by the use of these oils. Murine double minute 2 (MDM2) was found to interact well with chlorogenic acid and oleuropein, two compounds from coffee and olive oils, respectively. Coffee oil and olive oil were found to be promising therapeutic agents for MAL-induced testicular toxicity and oxidative damage.

## 1. Introduction

Malathion is an effective and widely used pesticide. In previous studies, MAL has been shown to have a negative impact on the reproductive system [1]. Cell development and differentiation may be disrupted by the generation of reactive oxygen species (ROS) and the induction of intracellular oxidative stress, which has been hypothesized to have multiple effects [2]. One of the most important causes of male infertility is oxidative stress. DNA fragmentation and lipid peroxidation disrupt spermatozoon ability to support normal embryonic development and the motility of its cells at the spermatozoon level. As a result, oxidative stress can disrupt the germinal epithelium ability to differentiate normal spermatozoa at this point in the development [3]. Steroidogenesis was inhibited, apoptosis in germ cells was induced, and the seminiferous epithelium grew in response to MAL [4]. Previous studies have shown that MAL lowers testosterone levels, inhibits acetylcholinesterase, damages DNA, reduces chromatin in spermatogonia and spermatids, and has a negative impact on male mouse reproductive abilities. Semen parameters were altered as a result of exposure to MAL at pubertal age [5,6]. Antioxidants safeguard the testes by keeping ROS levels below a key threshold in order to prevent testicular dysfunction [7].

Many diseases have been successfully treated with the help of medicinal plants. Medical herbs are known to be ideal for human health because they have fewer side effects than synthetic medications [8]. In addition to stimulating the nervous system, coffee oil has been shown to improve mood, alertness, and other psychoactive responses, neurological disorders, and metabolic syndrome conditions [9,10]. When it comes to the prevention of various diseases, olive oils are an essential ingredient in Mediterranean diets. In olive oil, the primary fatty acid is oleic acid, which is one of several monounsaturated fatty acids. Antioxidant properties are also provided by a few minor compounds in olive oil [11].

The MDM2 and MDMX homologs are the most important negative regulators of p53. When cells are under stress, MDM2 suppresses p53 activity; however, in order for p53 to respond, MDM2 must be inhibited. MDM2 and MDMX decrease p53 activity in normal cells, but in the event of cellular stress, they must also be inhibited so that p53 can respond. MDM2 and MDMX are known to interact and have many, non-redundant roles in p53 protein activity modulation [12]. The p53 protein, a key tumor inhibitor, is activated in response to a variety of cellular stressors in an effort to stop and repair damage that can lead to tumor formation. When p53 is activated in response to these stresses, it can have very significant cellular consequences, such as permanent cell cycle arrest and cell death. To avoid unintended pathological consequences, p53 must be tightly regulated. The protein activity of p53 is regulated by MDM2 and MDMX, which interact and play multiple roles, but their roles are not redundant [12]. The primary objective of this research was to determine whether coffee oil and olive oil could protect rats with MAL-induced testicular toxicity from oxidative stress.

## 2. Materials and Methods

### 2.1. Animal Groups and Treatments

Rats were handled and acclimated to the conditions as described by Al-Asmari et al. [13]. King Abdulaziz University Animal Care and Use Committee (ACUC) issued guidelines for conducting the experiments, which were followed. The experiments were conducted according to all the regulations, guidelines and rules, including EU Directive 2010/63/EU for practical research for animals. Randomly, six groups of rats were formed (*n* = 10 rats/group). Each group received the following therapy: A normal control group was used for the first study, while the second group was given MAL (100 mg per kg BW). The third and fourth groups were given coffee oil and olive oil in the same doses (400 mg per kg BW) in addition to the same dose of pesticide given to the second group; the fifth and sixth groups were given coffee oil and olive oil in the same doses as the third group for seven weeks.

### 2.2. Analysis of Blood Serum

Diethyl ether was used to anesthetize the animals after a 12-h fast [13]. After 49 days (7 weeks), the rats under study were subjected to a twelve-hour fasting period, with free access to water. Then, diethyl ether was given to them as an anesthetic drug. The venous plexus in the orbital region was used to collect the blood sample, which was then placed without heparin in centrifuge tubes. The tubes were then subjected to centrifugation at 2500 rpm for 15 min. After the centrifugation process, the serum was collected from the centrifuge tubes and put in storage at −80 °C. In addition, serum levels were determined employing the techniques explained in [14,15,16,17] where the serum levels of GSH, SOD, MAD, and CAT, respectively were evaluated.

### 2.3. Histopathological Examination

Testis tissues preparation, preservation, staining, and examination were conducted following the modified method of Al-Asmari et al. [18]. Photomicrographing of histological sections was applied at Alborg Laboratory, Jeddah, KSA using modern automated processors (Leica TP 1020 tissue processor) and the pictures were taken with an IntelliSite Ultra-Fast Scanner (Digital pathology slide scanner from Philips). Stained sections were examined for circulatory disturbances, inflammation, degenerations, apoptosis, necrosis, and any other pathological changes in the examined tissues.

### 2.4. Bioinformatics Analysis 

Selection of Enzymes for Docking Study

The method detailed by Atatreh et al. [19] was used for the selection of inhibitors of MDM2 and p53 interactions.

### 2.5. Molecular Docking Study

The crystal structures of MDM2 proteins (PDB ID: 6GGN) were obtained by downloading from the Research Collaboratory for Structural Bioinformatics (RCSB). The Pub-Chem database, as illustrated in the sources [11,20,21,22], was used to identify the top compounds in coffee oil and olive oil as shown in (Table 1).

Schrödinger Suite 2021-3, which includes Maestro, was used for molecular docking investigations. It was also used for ligand preparation and protein assembly, as well as active site prediction using interfaces such as LigPrep, Receptor grid generation (from Maestro), and SiteMap. The proteins and ligands were categorized as rigid during preparation. P53 and MDM2 protein-interaction inhibitors were redocked with MDM2 receptors for a second time to ensure that the protocol was still intact and effective.

### 2.6. Analysis of The Statistics

In this study, statistical analysis was conducted using IBM SPSS version 24 “Statistical Package for the Social Sciences”. The median and range of values, as well as any standard deviations connected with the data, are included in the results (SE). For example, when comparing Groups 2, 3, and 4, the primary focus of the work is on comparison. In order to compare the six groups, a statistical method known as one-way ANOVA was used. When the ANOVA revealed a statistically significant difference between groups, the least significant difference (LSD) was used following the ad hoc test. A significant *p*-value of less than 0.05 was achieved, as compared with other tests that did not achieve this.

## 3. Results

### 3.1. Assessment of Biochemical Markers

**Figure 1:**
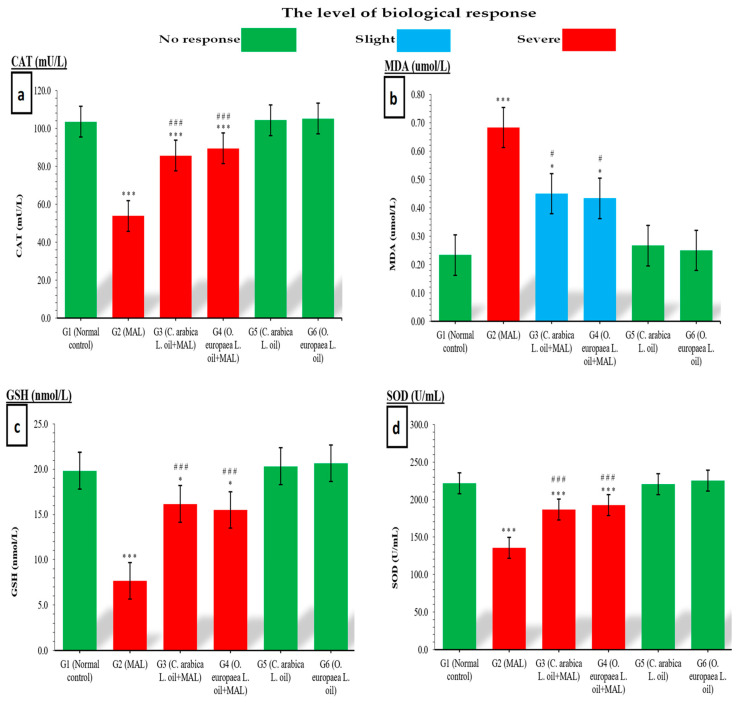
Influence of coffee (*C. arabica* L.) and olive (*O. europaea* L.) oils on serum (**a**) CAT, (**b**) MDA, (**c**) GSH and (**d**) SOD levels. The mean values obtained were at *p* 0.05 * and *p* 0.001 *** when the experimental groups were compared with the normal control group. When compared with the MAL-intoxicated rats, significance was also observed at *p* 0.05 ^#^ and *p* 0.001 ^###^.

Comparing the MAL-intoxicated rats (G2) with the control rats (G1), the serum levels of CAT (mU/L) were statistically lower (*p* ≤ 0.001) in the MAL-intoxicated rats (G2). When fed to MAL-poisoned rats at 400 mg/kg BW, coffee (G3) and olive (G4) oils a significantly reduced CAT level (*p* ≤ 0.001) was noted when compared with G1 rats (uninterfered control). A significant (*p* ≤ 0.001) improvement in the serum levels of CAT was observed in MAL-poisoned rats given 400 mg/kg BW of coffee(G3) and olive (G4) oils daily for a period of seven weeks when compared with (G2) (Figure 1a).

Malondialdehyde (mol/L) serum levels of MAL-poisoned G2 rats increased significantly (*p* ≤ 0.001) when compared with the serum MDA levels of unaffected G1 rats (the standard control group). Similarly, when MAL-poisoned rats were fed 400 mg/kg BW of coffee (G3) and olive (G4) oil daily, the serum levels of MDA increased significantly (*p* ≤ 0.05) when compared with G1 rats (the normal control). In a study involving MAL-poisoned rats, the administration of coffee and olive oils (G3 and G4) had a significant outcome of lower serum MDA levels (*p* ≤ 0.05) than those observed in the MAL-poisoned group (G2) (Figure 1b).

The levels of GSH (nmol/L) in the serum of MAL-poisoned rats (G2) decreased significantly (*p* ≤ 0.001) after 7 weeks of toxicity when compared with the levels in the serum of the normal control group. Moreover, serum GSH levels in MAL-poisoned rats were found to be significantly lower (*p* ≤ 0.05) when they were given daily doses of 400 mg/kg BW of the oils of coffee (G3) and olive (G4) when compared with the G1 rats. In MAL-poisoned rats, daily gavage with coffee oil (G3) and olive oil (G4) resulted in a significant increase (*p* ≤ 0.001) in GSH levels when compared with the MAL-poisoned rats (G2) (Figure 1c). 

MAL-intoxicated rats (G2) had significantly (*p* ≤ 0.001) lower SOD (U/mL) levels in their serum than normal control rats (G1). When comparing SOD levels in MAL-poisoned rats treated with the oils of coffee (G3) and olive (G4) at doses of 400 mg/kg BW to those in normal control rats, similar findings were observed (G2). Comparing the G3 (MAL-poisoned rats supplemented with coffee oil), and G4 groups (MAL-poisoned rats treated with olive oil) with the G2 group (MAL-intoxicated rats), a significant (*p* ≤ 0.001) elevation in the serum SOD levels was observed (Figure 1d).

### 3.2. Histopathological Studies

**Figure 2:**
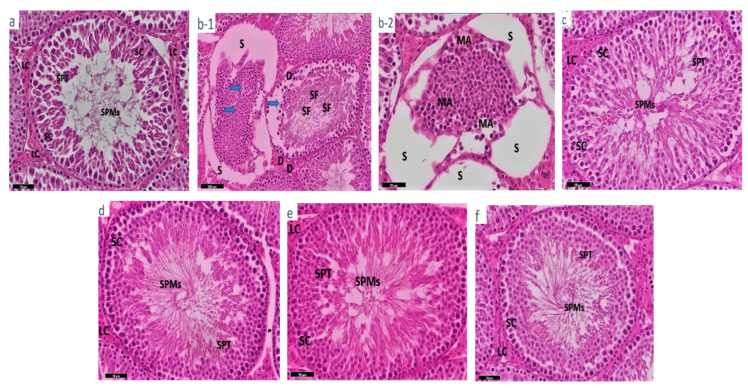
Photomicrograph of rat testis (G1–G6): Photo (**a**,**c**–**f**) seminiferous tubules containing mature sperms (SPMs) with Sertoli cells (SC), spermatids (SPT) and the Leydig cells (LC). Photo (**b-1**,**b-2**) showing spaces due to tubule shrinkage (S) with evidence of degeneration necrosis (thick blue arrow) and sperm fragmentation (SF), stromal destruction (D) and indicated arrested maturation, due to toxicity of MAL leading to a shrinkage (MA) in tubules, Leydig cells and Sertoli cells.

Histological sections from testis of normal control rats (Group 1) showed fine well-demarcated interstitial tissue and interstitial Leydig cells with tubules. These tissues were regular with no distortion and cells were well visualized, and blood supply capillaries and other blood vessels were intact. Interstitial Leydig cells showed chromatin details and prominent active nuclei. Seminiferous tubules were intact and surrounded with well-demarcated connective tissues with Sertoli cells and spermatids, even for wavy mature sperms (Figure 2a). Sections obtained from rats orally administrated with MAL (Group 2) exhibited detached spermatids, Sertoli cells and Leydig cells. There were noticeable features of sperm fragmentation and degeneration with a uniform cell’s maturation as evidence of toxic degeneration halting cell maturation. Features of cell interspacing and cytolysis with extreme necrotizing degeneration were also observed (Figure 2(b-1,b-2)).

Observing sections from (Group 3) rats that were orally administrated MAL and supplemented with coffee oil, the sections showed healthy cells, seminiferous tubules and cell lining. Moreover, the surrounding tubules were intact, and healthy Sertoli cells, Leydig cells, sperms, and myotics cells with well-preserved capillaries and a good blood supply similar to those of the normal control group (Figure 2c) were found. In (Group 4), which included rats orally treated with olive oil after MAL administration, the appearance of histological sections showed uniform and intact tissues and cells, including Sertoli cells, Leydig cells, sperms and myotic cells with minor capillary changes, but complete restoration. These changes were described as being similar to those in Group 3 (Figure 2d). In rats orally supplemented with coffee oil (Group 5) (Figure 2e), and olive oil (Group 6) (Figure 2f), the histological sections were normal and uniform with intact Sertoli cells, Leydig cells, sperms and myotic cells and tubules with completely normal architecture. The observable testis functioning cells exhibited euchromatin (well distributed), prominent nucleoli, with complete vitality, and healthy-looking architecture similar to that of the normal control group.

### 3.3. Molecular Docking Results

**Figure 3:**
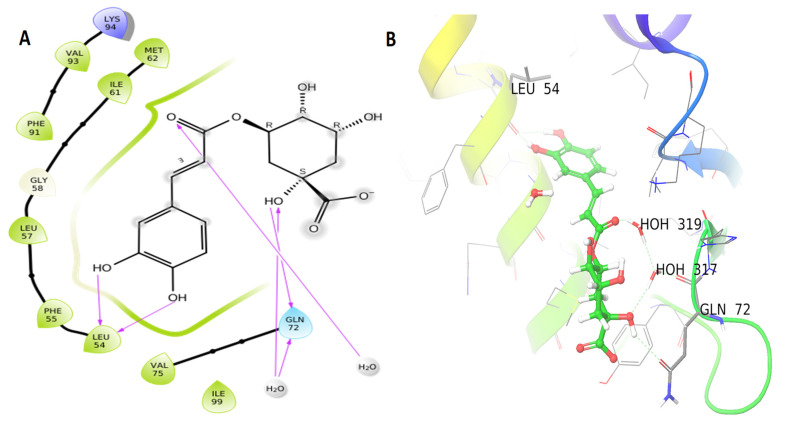
MDM2 protein interaction in 2D (**A**) and 3D (**B**), with ligands shown as balls in 3D, protein backbones represented as ribbons, and hydrogen bonds depicted as purple arrows. Chlorogenic acid 2D and 3D interactions are indicated in (**A**,**B**).

The docking results between MDM2 and the seven compounds identified in coffee oil that include chlorogenic acid, kahweol, cafestol, oleic acid, caffeine, linoleic acid, and palmitic acid (with binding energies of −7.564, −4.719, −4.584, −4.204, −4.024, −3.38, −2.895 kcal/mol, respectively) showed good binding energy to MDM2 (Table 1). However, chlorogenic acid to MDM2 had the highest binding energy among the seven binding energies in coffee oil. This compound interacts with four hydrogen bonds to the MDM2 protein: Gln 72 (two bonds) and Leu 54 (two bonds) (Figure 3A,B).

**Figure 4:**
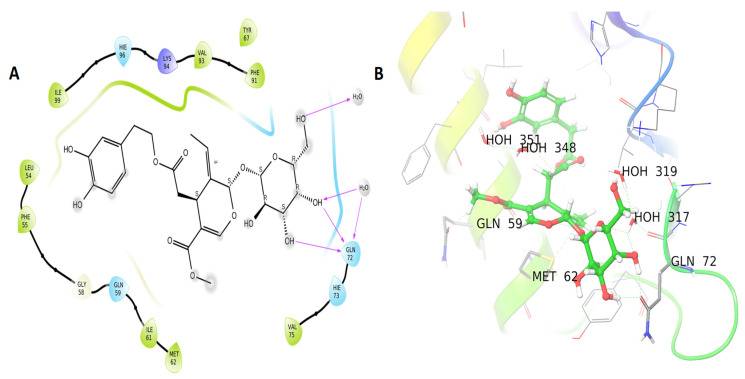
MDM2 protein interaction in 2D (**A**) and 3D (**B**): ligands shown as balls in 3D, protein backbones represented as ribbons, and hydrogen bonds depicted as purple arrows. Oleuropein 2D and 3D interactions are indicated in (**A**,**B**).

Table 1 presents the binding energies obtained by docking MDM2 with oleuropein, hydroxytyrosol, tyrosol, squalene, oleic acid, linoleic acid, stearic acid, and palmitic acid from olive oil (−7.652, −5.91, −4.474, −3.949, −3.909, −3.38, −3.317, and −3.263 k). According to the current findings the oleuropein molecule has a docking score of −7.652 kcal/mol and three hydrogen bonds with the MDM2 protein’s Gln 72 residues, (Figure 4A,B). The docking score of the attached ligand (P53 and MDM2 proteins-interaction-inhibitor) was −5.7 kcal/mol for the associated ligand.

## 4. Discussion

An organophosphate compound known as MAL is widely used in agriculture, veterinary medicine, public health, and food preparation and processing areas [23]. Although reproductive effects of malathion have yet to be fully understood, it has a wide range of toxicities [24]. Malathion raises MDA levels and decreases the activity of other antioxidant enzymes, which are all-important in preventing oxidative stress [25]. 

Understanding herb performance and effectiveness in improving health and quality of life is helping the public and medical professionals accept herbal medicine [26]. Arabica coffee has recently been found to contain a variety of antioxidants that have been shown to be effective in the process of treating multiple ailments [27]; while olives are regarded as a nutritious food because they contain phenolic compounds, which are known to have health benefits. Anti-inflammatory properties of phenolic compounds have been demonstrated in a number of recent studies [28,29].

In the present study the administration of MAL resulted in statistical decreases in serum GSH, CAT, and SOD levels, while significantly increasing serum MDA levels. According to previous studies, the oxidative damage caused by MAL and other pesticides can be seen in the reduction of GSH, CAT, and SOD levels in the serum and an increase in MDA concentrations [30,31]. Toxic exposure to various organophosphates can lead to an increase in the generation of free radical attack, which can induce cholinesterase inhibition and oxidative stress. Oxidative defense mechanisms, such as SOD and CAT, protect cells from free radical damage. GSH, an antioxidant, aids in the removal of toxins from the kidneys by aiding in their dissolution and expulsion. When the body’s ability to combat oxygen radicals is out of balance, oxidative stress occurs [32].

Coffee oil and olive oil were found to reduce the physiological effects of MAL intoxication in rats. As a result, these oils were found to be effective in protecting the experimental animals from MAL toxicity. Statistically significant increases in GSH, CAT, and SOD levels were also observed, while MDA levels were dramatically reduced after administration of coffee and olive oils. Cafestol and kahweol, two compounds found in coffee oil, may also cause the synthesis of GSH, a nutrient important for detoxification and liver protection [33]. According to Al-Megrin et al. [27], the antioxidant markers GSH, SOD, and catalase were significantly increased in diabetic male rats after green coffee treatment.

One explanation for low levels of SOD and CAT may be due to increased ROS production in OP poisoning. Oxidative stress and ROS scavenging by oleuropein increased the activity of SOD and CAT in rats, which suggests that oleuropein can repair and maintain these enzymes [34]. Olive oil had a protective effect against the toxicological effects of various chemical pollutants, suggesting that it plays a function in free reactionary antioxidant defense systems against their lethality [35,36]. When used as protective agents, the phytochemical constituents of the coffee and olive oils studied may prevent MAL from activating into a reactive state. Prevention of the oxidation process of substrates in the cell can be done by taking in antioxidants in the diet. As a result, incorporating antioxidants into our diet can help prevent oxidative damage related chronic diseases [37]. Preservation of food is also achieved through the application of antioxidants which play a critical role of helping to inhibit lipid oxidation [38].

Detached spermatids, Sertoli cells, and Leydig cells were found in MAL-treated rats’ testicles in the current study. As evidence of toxic degeneration stopping cell maturation, there were also visible features of sperm fragmentation and degeneration with a uniform cell maturation. Cell interspacing and cytolysis with severe necrosis and apoptosis were also found. Malathion exposure has been linked to similar degenerative changes in the testes [24,39]. Coffee oil and olive oil were found to have a positive effect on the pathological changes caused by MAL intoxication in rats. Seminiferous tubules, cell lining, intact and healthy Sertoli cells, Leydig cells, sperms and myotics cells with well-preserved capillaries and good blood supply were found in the testis section. Compared with the MAL-intoxicated group, the testicular tissues and cells had returned to normal under the assumption of the protective effects of coffee and olive oils. The outcomes of this investigation were consistent with those of Al-Megrin et al. [27], Bhardwaj et al. [39], and Khayyat, [40].

MDM2 blocks p53 activity by inhibiting its transcriptional activity [41], by ubiquitin ligase activity that promotes p53 proteasomal hydrolysis or by interacting with P53 and causing its expulsion from the nucleus [42]. As a result of MDM2 overexpression in cancer cells, p53 is suppressed and does not stimulate the production of apoptosis and cell cycle arrest genes. Because of its critical function in the activation of apoptosis and cancer cell death, novel inhibitors of MDM2 and p53 binding are urgently required [43]. Two constituents from the oils of coffee (chlorogenic acid) and olive (oleuropein) showed good interaction with the MDM2 (6GGN) enzyme. Chlorogenic acid and oleuropein have been shown in other studies to protect testicular cells from oxidative damage and cancer cell growth [44,45]

## 5. Conclusions

This study illustrates uniquely that MAL-induced reproductive dysfunctions in male Albino rats can be alleviated by the use of olive and coffee oils. The presence of the antioxidant agents in the plants play a crucial role in the bioactive processes of MAL toxicity protection. Coffee and olive oils have been shown to protect against MAL-induced testis injury, but further research is needed to better understand the mechanisms of this protection. Furthermore, computer simulations showed that chlorogenic acid (found in coffee oil) and oleuropein (found in olive oil) both inhibit MDM2 (6GGN). Our findings need to be confirmed in vivo, which necessitates more research and extensive testing of secondary molecules in the clinical setting.

## Figures and Tables

**Table 1:** XP GScore, glide energy, and glide model of coffee oil and olive oil compounds on MDM2 (6GGN).

	Name	PubChem CID	XP GScore	Glide Energy	Glide Emodel
MDM2 (6GGN)	coffee oil compounds				
	Chlorogenic acid	1794427	−7.564	−36.496	−49.551
	Kahweol	114778	−4.719	−22.304	−26.808
	Cafestol	108052	−4.584	−25.19	−17.839
	Oleic acid	445639	−4.204	−31.943	−34.117
	Caffeine	2519	−4.024	−24.182	−30.139
	Linoleic acid	5280450	−3.38	−34.192	−33.195
	Palmitic acid	985	−2.895	−26.24	−30.925
	olive oil compounds				
	Oleuropein	5281544	−7.652	−44.215	−54.288
	Hydroxytyrosol	82755	−5.91	−21.452	−22.75
	Tyrosol	10393	−4.474	−18.42	−19.683
	Squalene	638072	−3.949	−33.026	−35.897
	Oleic acid	445639	−3.909	−32.642	−33.767
	Linoleic acid	5280450	−3.38	−34.192	−33.195
	Stearic acid	5281	−3.317	−31.351	−34.989
	Palmitic acid	985	−3.263	−27.325	−29.101
Control	P53 and MDM2 Protein-Interaction-Inhibitor	17754765	−5.7	−16.3	−14.4

## Data Availability

All relevant data are within the manuscript. All data were statisti-cally analyzed, as mentioned in the submitted manuscript.
